# Comparison of Dydrogesterone and Medroxyprogesterone in the Progestin-Primed Ovarian Stimulation Protocol for Patients With Poor Ovarian Response

**DOI:** 10.3389/fendo.2021.708704

**Published:** 2021-09-24

**Authors:** Junwei Zhang, Mingze Du, Zhen Li, Wenxia Liu, Bingnan Ren, Yuchao Zhang, Yichun Guan

**Affiliations:** The Reproductive Center, The Third Affiliated Hospital of Zhengzhou University, Zhengzhou University, Zhengzhou, China

**Keywords:** dydrogesterone, medroxyprogesterone, progestin-primed ovarian stimulation, poor ovarian responder, controlled ovarian hyperstimulation

## Abstract

**Objective:**

To compare the clinical outcomes of dydrogesterone (DYG) and medroxyprogesterone (MPA) in the progestin-primed ovarian stimulation (PPOS) protocol for patients with poor ovarian response (POR).

**Patients and Methods:**

This was a retrospective cohort study. Women with POR who underwent IVF/ICSI at the Reproductive Center of Third Affiliated Hospital of Zhengzhou University between January 2020 and January 2021 were included. The primary outcome measure of our study was the number of oocytes retrieved. The secondary outcome measures in the present study were the number of 2PN, number of available embryos, oocyte retrieval rate, fertilization rate, viable embryo rate per oocyte retrieved, cancellation rate and pregnancy outcomes of the first embryo transfer cycle, including the biochemical pregnancy, clinical pregnancy and miscarriage rates.

**Results:**

In total, 118 women underwent hMG +DYG protocols, and 692 women who underwent hMG +MPA met the Bologna criteria for POR. After baseline characteristics were balanced using the PSM model, 118 hMG +DYG protocols were matched to 118 hMG +MPA protocols, and the baseline characteristics were comparable between the two groups. The numbers of oocytes retrieved, 2PN, and available embryos and the oocyte retrieval rate, fertilization rate, viable embryo rate per oocyte retrieved and cancellation rate of the hMG+DYG and hMG+MPA protocols were comparable. Altogether, 66 women in the hMG+DYG group and 87 women in the hMG+MPA group underwent first embryo transfers. In the hMG+DYG group, 81.8% (54/66) of the patients underwent cleavage embryo transfers; similarly, 79.3% (69/87) of patients in the hMG+MPA group had cleavage embryo transfers (P=0.70).The biochemical pregnancy rate of the hMG+DYG group was 42.4%, and this was comparable to the rate in the hMG+DYG group, at 34.5% (P=0.32). The clinical pregnancy rates were similar between the two groups (36.4% *vs.* 31.0%, P=0.49), and there was no significant difference in the rate of miscarriage between the two groups (12.5% *vs.* 29.6%, P=0.14).

**Conclusion:**

For women with POR, the clinical outcome of the hMG + DYG group was similar to that of the hMG + MPA group, indicating that both combinations can be useful options for PPOS protocols.

## Introduction

Infertility affects approximately 10% of reproductive-aged couples worldwide (1). *In vitro* fertilization (IVF) has become the most effective treatment for infertility caused by tubal or other factors as well as for unexplained infertility (2). Controlled ovarian hyperstimulation (COH), which suppresses the luteinizing hormone (LH) surge, increases the efficacy of treatment by promoting the maturation of multiple oocytes and availability of embryos for transfer, making COH a crucial step in IVF (3). In routine clinical practice with COH, up to 9%~24% of patients have poor ovarian response (POR) (4). POR is characterized mainly by a number of follicles that develop during COH below the intended target, a high level of gonadotropin (Gn), a high cycle cancellation rate, low numbers of harvested oocytes and available embryos, and a low pregnancy rate and live birth rate (LBR). At present, the Bologna criteria, discussed and formulated by the European Society of Human Embryology and Reproduction and the American Society of Reproductive Medicine in 2011, are the most widely used standards in clinical practice.

The progestin-primed ovarian stimulation (PPOS) protocol, a new ovarian protocol proposed by Dr Yanping Kuang in 2015, has advantages in terms of its effectiveness for suppressing the LH surge as well as its oral administration (5). The PPOS protocol uses progestin combined with exogenous gonadotropin, and the appropriate progestin is crucial to the success of the PPOS protocol. Progesterone may regulate the LH peak at AVPV and ARC sites in the hypothalamus. PPOS uses progesterone to resist the positive feedback effect and avoids its auxiliary positive feedback (6, 7). Medroxyprogesterone (MPA) was the first progestin to be used to suppress the premature LH surge, and its effectiveness and safety have been proven (5, 8–10). Dydrogesterone (DYG), the structure of which is most similar to that of natural progesterone, has no estrogenic, androgenic, antiandrogenic, or glucocorticoid activity and is used mainly in the hormone replacement cycle, for corpus luteum support and in cases of threatened abortion (11). A randomized controlled trial (RCT) including 516 first IVF/ICSI cycles of women with normal ovarian reserve compared DYG and MPA in the PPOS protocol, and the results showed that DYG can be used as an appropriate alternative progestin in a PPOS protocol (12). Another study compared the use of DYG and MPA in women with polycystic ovarian syndrome (PCOS) and showed that DYG could achieve oocyte retrieval and pregnancy outcomes comparable to those of MPA (13). However, the use of DYG as an alternative to MPA and as an appropriate progestin in the PPOS regimen has yet to be explored in women with POR. Therefore, the purpose of this study was to compare the application of DYG *versus* MPA in the PPOS protocol of women with POR.

## Materials and Methods

### Study Design and Population

This was a retrospective cohort study approved by the review board of the Third Affiliated Hospital of Zhengzhou University. For this study, we included women with POR who underwent IVF/ICSI at the Reproductive Center of Third Affiliated Hospital of Zhengzhou University from January 2020 to January 2021. Women aged ≤ 45 years were included, and all patients were diagnosed with POR according to the Bologna criteria definition by the European Society of Human Embryology and Reproduction and the American Society of Reproductive Medicine. At least two of the following three features were present: (i) advanced maternal age (≥40 years) or any other risk factor for POR; (ii) previous POR (≤3 oocytes with a conventional stimulation protocol); and (iii) an abnormal ovarian reserve results (antral follicle count (AFC), 5–7 follicles or AMH, 0.5 –1.1 ng/ml) (14). We excluded cycles with endometriosis grade 3 or higher, uterine malformations, endometrial polyps, preimplantation genetic testing (PGT) or donor oocytes. Furthermore, cycles with incomplete records for certain parameters, such as basal AFC or AMH, were excluded.

### Controlled Ovarian Hyperstimulation Protocols

For the PPOS protocol, COH was initiated on the second or third day of the menstrual cycle (MC). Patients were given oral 20 mg DYG (Abbott Biologicals B.V., Netherlands) or 6 mg of MPA (Beijing Zhong Xin Pharmaceutical, China) combined with human menopausal gonadotropin (hMG) (Anhui Fengyuan Pharmaceutical, China) at a dose of 150 to 300 IU/day depending on maternal age, body mass index (BMI), AMH and AFC. Then, follicle growth was monitored by vaginal ultrasound combined with serum hormone analysis 3-5 days later. If necessary, the dose of hMG was adjusted according to follicle development. When the diameter of the dominant follicle was greater than 20 mm or when at least three follicles reached 18 mm, the final stage, namely, the ovulation trigger, was performed with triptorelin (100 μg) (Ferring International Center SA, Germany) and 2000 IU of human chorionic gonadotropin (hCG) (Lizhu Pharmaceutical Trading, China), followed by oocyte pickup 36 hours later. Based on the sperm quality, conventional IVF or ICSI was performed.

Serum hormones, including LH, E2, and P levels on MC2-3, MC5-8, MC9-11, the trigger day and the day after trigger, were analyzed using the Roche Cobas immunoassay (Roche Diagnostics, Germany).

### Embryo Transfer and Endometrial Preparation Protocols

Whole embryos were frozen. Endometrial preparation for FET was performed in natural cycles, artificial/induced ovulation cycles and downregulation + artificial cycles for women with regular menstrual cycles plus spontaneous ovulation, irregular menstrual cycles, and endometriosis, respectively. Follicle and endometrial scanning was performed by vaginal ultrasound, and one to two embryos or one blastocyst transplantation was performed using abdominal ultrasound after 3 or 5 days of endometrial development with luteosterone. Routine corpus luteum support, namely, oral DYG (2 times daily, 10 mg once) (Abbott Co. America) and intravaginal administration of 90 mg of a progesterone sustained-release vaginal gel (Merck Co. Germany), was given. If pregnancy occurred, corpus luteum support was continued at least until 55 days after transplantation. All data were obtained by reviewing our reproductive center’s medical records.

### Outcome Measures and Definition

The outcome measures in the present study included the number of oocytes retrieved, number of 2PN, number of available embryos, oocyte retrieval rate, fertilization rate and viable embryo rate per oocyte retrieved. We also analyzed the cancellation rate, defined as no viable embryo *via* oocyte retrieval. The preterm LH surge was also analyzed, defined as a serum LH level greater than 10 IU/L before the trigger day. The pregnancy outcomes of the first embryo transfer cycle, including the biochemical pregnancy rate (defined as serum β-hCG ≥ 50 IU/L), clinical pregnancy rate (diagnosed by ultrasonographic visualization of one or more gestational sacs or definitive clinical signs of pregnancy), the clinically documented ectopic pregnancy, especially miscarriage rates (spontaneous loss of a clinical pregnancy before 22 completed weeks of gestation) were also analyzed (15).

### Statistical Analysis

A prospective score matching (PSM) model was applied to balance the baseline characteristics between the hMG +DYG group and hMG +MPA group, including maternal age, BMI, duration of infertility, type of infertility (primary/secondary infertility), infertility diagnosis (POR/POR +tubal factor/POR + male factor/POR + others), basal serum FSH level, basal serum LH level and AFC. The propensity score was obtained from a logistic regression model. Patients using DYG were matched with the patients using MPA at a 1:1 ratio based on the propensity score with a standard caliper width of 0.2.

The normality of continuous data was checked by the one-sample Kolmogorov-Smirnov test. Continuous variables are expressed as the mean ± SD, and Student’s t-test or the Wilcoxon rank-sum test was used to assess between-group differences properly. Categorical variables are represented as the number of cases (n) and percentage (%). The means from chi-square analyses were used to assess the differences between groups with Fisher’s exact test when necessary.

All statistical management and analyses were performed using SPSS software, version 24.0. A two-sided P value <0.05 was considered statistically significant.

## Results

### Study Population

A total of 118 women underwent hMG +DYG protocols, and 692 women who underwent hMG +MPA met the Bologna criteria for POR from January 2020 to January 2021. After balancing the baseline characteristics using the PSM model, 118 hMG +DYG protocols were matched with 118 hMG +MPA protocols.

### Baseline Characteristics

After postmatching analysis, the baseline characteristics, including maternal age, paternal age, BMI, duration of infertility, type of infertility, indication for IVF/ICSI, basal serum FSH level, basal serum LH, AMH and AFC, were comparable between the hMG +DYG group and hMG +MPA group. A detailed comparison between the groups is shown in **Table 1**.

**Table 1:** Baseline characteristics of women treated with the hMG+DYG and hMG+MPA protocols.

	hMG+DYG (n=118)	hMG+MPA (n=118)	P value
Maternal age (years)	35.7 ± 5.8	35.8 ± 5.9	0.91
Paternal age (years)	35.7 ± 6.2	36.3 ± 6.8	0.50
Body mass index (kg/m2)	23.7 ± 2.9	24.4 ± 2.8	0.06
Duration of Infertility (years)	4.4 ± 2.0	4.0 ± 2.5	0.52
Type of infertility			0.90
Primary infertility	49/118 (41.5)	48/118 (40.7)	
Secondary infertility	69/118 (58.5)	70/118 (59.3)	
Indication of IVF/ICSI			0.44
POR	18/118 (15.3)	18/118 (15.3)	
POR +Tubal factor	26/118 (22.0)	36/118 (30.5)	
POR + Male factor	46/118 (39.0)	43/118 (36.4)	
POR + Others	28/118 (23.7)	21/118 (17.8)	
Basal serum FSH level (IU/l)	9.5 ± 4.9	9.2 ± 4.0	0.53
Basal serum LH level (IU/l)	5.5 ± 3.2	5.9 ± 3.5	0.54
AMH (ng/ml)	0.9 ± 0.8	0.9 ± 0.7	0.86
Basal antral follicle count	5.4 ± 3.4	5.8 ± 3.5	0.57

Data are presented as the mean ± SD for continuous variables and n (%) for categorical variables.

### Cycle Characteristics

**Table 2** presents the detailed cycle characteristics of women treated with the hMG+DYG and hMG+MPA protocols. The dosage of gonadotropins (2728.6 ± 873.9 *vs* 2715.7 ± 926.3, P=0.91) and duration of ovarian stimulation (10.0 ± 2.7 *vs* 9.8 ± 2.7, P=0.60) between groups were comparable. The rate of IVF was similar between groups (60.2% *vs* 58.5%, P=0.79). The numbers of oocytes retrieved, 2PN, and available embryos and the oocyte retrieval rate, fertilization rate, viable embryo rate per oocyte retrieved and cancellation rate were comparable between the hMG+DYG and hMG+MPA protocols.

**Table 2:** Cycle characteristics of women treated with the hMG+DYG and hMG+MPA protocols.

	hMG+DYG (n=118)	hMG+MPA (n=118)	P value
Dosage of gonadotropins (IU)	2728.6 ± 873.9	2715.7 ± 926.3	0.91
Duration of ovarian stimulation (days)	10.0 ± 2.7	9.8 ± 2.7	0.60
Fertilization method			0.79
IVF	71/118 (60.2)	69/118 (58.5)	
ICSI	47/118 (39.8)	49/118 (41.5)	
LH values on the trigger day (IU/l)	5.2 ± 4.5	4.4 ± 3.3	0.11
E2 values on the trigger day (pg/ml)	1304.4 ± 940.1	1542.2 ± 1080.2	0.07
P values on the trigger day (ng/ml)	0.5 ± 0.6	0.5 ± 0.5	0.55
Premature LH surge rate (%)	3/118 (2.5)	2/118 (1.7)	0.65
No. of oocytes retrieved	3.9 ± 2.8	4.5 ± 3.0	0.10
No. of 2PN	2.7 ± 2.0	2.8 ± 2.2	0.95
No. of available embryos	2.1 ± 1.7	2.3 ± 2.0	0.45
Oocyte retrieved rate (%)	74.4 ± 16.8	71.0 ± 20.0	0.14
Fertilization rate (%)	56.9 ± 21.5	55.5 ± 23.7	0.26
Viable embryo rate per oocyte retrieved (%)	50.5 ± 24.0	46.3 ± 24.2	0.16
Cancellation rate (%)	20/118 (16.9)	26/118 (22.0)	0.32

Data are presented as the mean ± SD for continuous variables and n (%) for categorical variables.

### Hormone Profile

**Figure 1:**
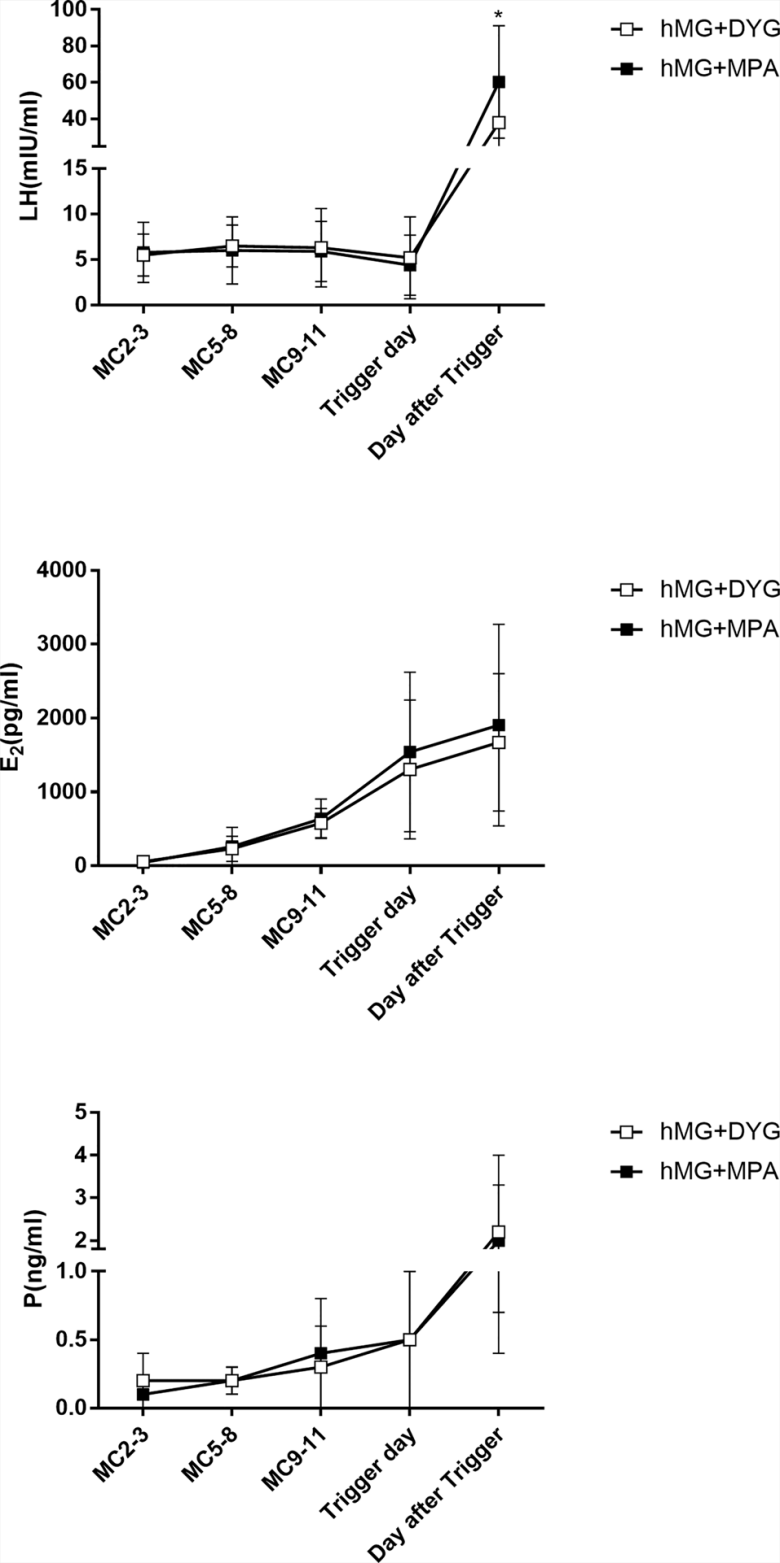
Serum hormone profiles of the hMG+DYG and hMG+MPA protocols. The asterisk indicates significant changes in the values at different time points between the two groups (*P < 0.001).LH,luteinizing hormone; E2, estrogen; P, progesterone.

The detailed hormone profiles (LH, E2 and P) during COH are shown in **Figure 1**. Regarding serum LH, the concentrations of LH were controlled only by DYG or MPA. Three cases in the hMG+DYG group and two cases in the hMG+MPA group experienced a premature LH surge (LH>10 IU/L) during COH (2.5% *vs.* 1.7%, P=0.65). No significant differences in LH levels were observed on MC2-3 (5.5 ± 2.3 *vs.* 5.8 ± 3.3, P=0.57), MC5-8 (6.5 ± 2.3 *vs.* 6.0 ± 3.7, P=0.75), MC9-11 (6.3 ± 4.3 *vs.* 5.9 ± 3.3, P=0.39) or the trigger day (5.2 ± 4.5 *vs.* 4.4 ± 3.3, P=0.11). However, the serum The LH level was significantly higher in the hMG+MPA group than in the hMG+DYG group on the day after triggering (38.1 ± 21.4 *vs.* 60.3 ± 30.8, P<0.01). In other words, after the trigger, the LH level of the MPA group rose more significantly than that of the DYG group.

Regarding serum E2, E2 gradually increased with the development of multiple follicles, and at each observation point, the E2 levels of the hMG+DYG and hMG+MPA groups were similar. The serum P gradually increased from MC2-3, and there were no statistically significant differences between groups at each observation point. Because our center did not perform routine testing of FSH levels during the period of ovulation induction, this study did not analyze the changes in serum FSH levels.

### The Pregnancy Outcomes

A total of 66 women in the hMG+DYG group and 87 women in the hMG+MPA group underwent first embryo transfer. Twenty-five percent of women in the hMG+DYG group underwent single embryo or single blastocyst transfer, which is comparable to the 28.7% in the hMG+MPA group. A total of 81.8% of women in the hMG+DYG group and 79.3% of women in the hMG+MPA group underwent cleavage-stage embryo transfer. The endometrial preparation protocols and endometrial thickness were similar between the two groups. The biochemical pregnancy rate of the hMG+DYG group was 42.4%, which was comparable to the 34.5% of the hMG+DYG group (P=0.32). The clinical pregnancy rate was similar between the two groups (36.4% *vs.* 31.0%, P=0.49). There was no significant difference in the rate of miscarriage between the two groups (12.5% *vs.* 29.6%, P=0.14). The detailed pregnancy outcomes are shown in **Table 3**.

**Table 3:** Cycle characteristics and pregnancy outcomes of the first embryo transfer cycle.

	hMG+DYG	hMG+MPA	P value
No. of cycles	66	87	
No. of embryos transferred per cycle (%)			0.68
Single	17/66 (25.8)	25/87 (28.7)	
Double	49/66 (74.2)	62/87 (71.3)	
Embryo stage at transfer (%)			0.70
Cleavage stage	54/66 (81.8)	69/87 (79.3)	
Blastocyst stage	12/66 (18.2)	18/87 (20.7)	
Endometrial preparation protocols			0.66
Natural cycles	30/66 (45.5)	38/87 (43.7)	
Artificial cycles	18/66 (27.3)	31/87 (35.6)	
Induced ovulation cycles	3/66 (4.5)	4/87 (4.6)	
Down-regulation + artificial cycles	15/66 (22.7)	14/87 (16.1)	
Endometrial thickness (mm)	9.4 ± 1.6	9.3 ± 2.0	0.76
Biochemical pregnancy rate	28/66 (42.4)	30/87 (34.5)	0.32
Clinical pregnancy rate	24/66 (36.4)	27/87 (31.0)	0.49
Miscarriage rate	3/24 (12.5)	8/27 (29.6)	0.14

Data are presented as the mean ± SD for continuous variables and n (%) for categorical variables.

## Discussion

In this study, DYG and MPA were used in PPOS for women with POR diagnosed per the Bologna criteria. The numbers of oocytes retrieved, 2PN, and available embryos and the pregnancy outcomes of the first embryo transfer were comparable between the two groups, suggesting that DYG was the appropriate alternative progestin for the PPOS protocol.

To our knowledge, regarding the comparison of DYG and MPA in PPOS protocols, there were only two studies, all from the same center in China. One is a RCT that included 516 patients with normal ovarian reserve and compared DYG 20 mg/day with MPA 10 mg/day (12). There was no significant difference in the number of oocytes retrieved (10.8 ± 6.3 *vs.* 11.1 ± 5.8, P = 0.33) or viable embryo rate per oocyte retrieved (37.4% *vs.* 35.6%, P= 0.16) between groups. By analyzing the hormone levels during COH, the mean LH level in the hMG + DYG group was always higher than that in the hMG + MPA group (P < 0.001). Another study was a retrospective cohort study performed in 2019, including 420 PCOS patients who underwent DYG (n=105) or MPA (n=315). The dosage of DYG was 20 mg/day, and the dosage of MPA was 10 mg/day. The numbers of oocytes retrieved in the two protocols were similar (16.1 ± 6.5 *vs* 15.1 ± 10.0, P=0.342). In the hMG + DYG group, the mean LH levels were significantly higher than those in the hMG + MPA group on cycle days 9–11 and the trigger day, and the dose of hMG was significantly lower (1710.7 ± 431.6 *vs* 1891.3 ± 402.2 IU, P<0.001) (13).

Consistent with previous research, the clinical outcome of the hMG+DYG group was similar to that of the hMG + MPA group, and both achieved satisfactory suppression of the LH surge, indicating that both combinations can be useful options for PPOS protocols. The numbers of oocytes retrieved, 2PN, and available embryos and the oocyte retrieval rate, fertilization rate, viable embryo rate per oocyte retrieved and cancellation rate of the hMG+DYG and hMG+MPA protocols were comparable. Regarding the hormone levels during COH, in the POR population, the LH and E2 levels of the two groups were similar on MC2-3, MC5-8, MC9-11 and the trigger day. However, the serum LH level was significantly higher in the hMG+MPA group than in the hMG+DYG group on the day after triggering. This finding is different from those of previous studies. In the POR population of this study, a deeper inhibition of LH by MPA was not observed; the two groups of hMG dosages were similar, and the LH level of the MPA group rose more significantly than that of the DYG group after trigger. The specific biological mechanism of the phenomenon is not clear. We think that this phenomenon may be related to the half-lives of the different drugs. It is possible that different progestins have different binding affinities to progesterone receptors, leading to different hormone levels, and thus, their biological effects differ (16). Using the gonadotropin-releasing hormone agonist (GnRH-a) trigger alone may lead to a poor response by the hypothalamo-pituitary-ovarian (HPO) axis, and current clinical studies have shown that for the PPOS protocols, a double trigger, including GnRH-a and a low dose of hCG, can avoid the low response of the HPO axis, which may improve oocyte maturation (12, 17). In this study, a double trigger, namely, triptorelin (100 μg) plus 2000 IU of hCG, was used; therefore, the difference in the increase in LH level after the trigger in the two protocols may not affect the number of oocytes or maturation. However, more related experimental studies are needed. The pregnancy outcomes of the first embryo transfer were comparable between the two groups. This study shows the effectiveness of DYG in the POR population and is the appropriate alternative progestin for the PPOS protocol.

To the best of our knowledge, our study is the first to compare the application of DYG and MPA in women with POR. Diagnosis, treatment and fertility assistance for patients with POR have always been the focus of the field of reproduction, but these issues continue to be challenging. As the critical step of IVF, it is very important to choose suitable COH protocols, as this is key to the success rate. It has been reported that GnRH antagonist protocols have a 0.34% to 8.0% chance of failing to control the LH surge, and increased age and diminished ovarian reserve are the main risk factors (18–21). Since Kuang et al. (5) proposed the PPOS program in 2015. PPOS protocols are widely used in the POR population and can effectively suppress the LH surge and obtain reliable clinical outcomes (10, 22, 23). Choosing the appropriate progestin is very important for ensuring a high success rate for PPOS protocols. However, this study also has certain limitations. First, this was a retrospective cohort study, and there was interference from confounding factors. However, to reduce the influence of important confounding factors, this study used a PSM model to balance the baseline characteristics between the hMG +DYG group and hMG +MPA group. Second, due to the short period of time for hMG +DYG application in our center, the study did not analyze the LBR or cumulative live births of the two protocols. In our future research, this will be further explored.

## Conclusion

In conclusion, for women with POR, the clinical outcome of hMG + DYG was similar to that of hMG + MPA, and both achieved satisfactory suppression of the LH surge, indicating that these combinations can be useful options for PPOS protocols. Comparison of the effectiveness and safety of these two progestins requires further randomized controlled studies with large samples.

## Data Availability Statement

The raw data supporting the conclusions of this article will be made available by the authors, without undue reservation.

## Ethics Statement

The studies involving human participants were reviewed and approved by The review board of the Third Affiliated Hospital of Zhengzhou University. Written informed consent for participation was not required for this study in accordance with the national legislation and the institutional requirements.

## Author Contributions

JZ, MD, and YG designed the study and selected the population to be included and excluded. JZ and ZL were involved in the data extraction and analysis. BR and YZ reviewed the data. MD and JZ were involved in drafting this article. All authors contributed to the article and approved the submitted version.

## Conflict of Interest

The authors declare that the research was conducted in the absence of any commercial or financial relationships that could be construed as a potential conflict of interest.

## Publisher’s Note

All claims expressed in this article are solely those of the authors and do not necessarily represent those of their affiliated organizations, or those of the publisher, the editors and the reviewers. Any product that may be evaluated in this article, or claim that may be made by its manufacturer, is not guaranteed or endorsed by the publisher.
